# Constructing a Reward-Related Quality of Life Statistic in Daily Life—a Proof of Concept Study Using Positive Affect

**DOI:** 10.3389/fpsyg.2017.01917

**Published:** 2017-11-02

**Authors:** Simone J. W. Verhagen, Claudia J. P. Simons, Catherine van Zelst, Philippe A. E. G. Delespaul

**Affiliations:** ^1^Department of Psychiatry and Neuropsychology, Faculty of Health Medicine and Lifesciences, Maastricht University, Maastricht, Netherlands; ^2^GGzE Institute of Mental Health Care Eindhoven and De Kempen, Eindhoven, Netherlands; ^3^Department of Adult Psychiatry, Mondriaan Mental Health Trust, Heerlen, Netherlands

**Keywords:** quality of life, experience sampling, reward, monte carlo simulations, behavior setting

## Abstract

**Background:** Mental healthcare needs person-tailored interventions. Experience Sampling Method (ESM) can provide daily life monitoring of personal experiences. This study aims to operationalize and test a measure of momentary reward-related Quality of Life (rQoL). Intuitively, quality of life improves by spending more time on rewarding experiences. ESM clinical interventions can use this information to coach patients to find a realistic, optimal balance of positive experiences (maximize reward) in daily life. rQoL combines the frequency of engaging in a relevant context (a ‘behavior setting’) with concurrent (positive) affect. High rQoL occurs when the most frequent behavior settings are combined with positive affect or infrequent behavior settings co-occur with low positive affect.

**Methods:** Resampling procedures (Monte Carlo experiments) were applied to assess the reliability of rQoL using various behavior setting definitions under different sampling circumstances, for real or virtual subjects with low-, average- and high contextual variability. Furthermore, resampling was used to assess whether rQoL is a distinct concept from positive affect. Virtual ESM beep datasets were extracted from 1,058 valid ESM observations for virtual and real subjects.

**Results:** Behavior settings defined by Who-What contextual information were most informative. Simulations of at least 100 ESM observations are needed for reliable assessment. Virtual ESM beep datasets of a real subject can be defined by Who-What-Where behavior setting combinations. Large sample sizes are necessary for reliable rQoL assessments, except for subjects with low contextual variability. rQoL is distinct from positive affect.

**Conclusion:** rQoL is a feasible concept. Monte Carlo experiments should be used to assess the reliable implementation of an ESM statistic. Future research in ESM should asses the behavior of summary statistics under different sampling situations. This exploration is especially relevant in clinical implementation, where often only small datasets are available.

## Introduction

Mental health care is becoming more patient-centered. Every person is unique and the current classification systems miss relevant nuances for customized therapeutic interventions (Evans et al., 2013; McGorry and van Os, 2013). van Os (2014) argued to innovate assessment by making it more person-tailored and actively involve patients in the process. Clinicians should focus less on group characteristics and more on the individual's daily adaptation strategies and need for care (van Os, 2014). Over the years, diagnostic procedures have become more time consuming. Results are often complex latent structures that are not transparent and do not facilitate a collaborative communication between clinician and patient (van Staden, 2003; van Os, 2014). The main purpose of mental health care is to improve functioning as well as quality of life, in an empowering way. Most psychological interventions require motivation and engagement. An alienating communication does not help to engage patients in treatment. The ultimate goal is to assist patients in becoming more resilient, improve autonomy, reduce the impact of mental illness and improve well-being.

Resilient individuals are able to reduce their vulnerability by reducing the impact of symptoms and complaints in daily life. The reference point for therapeutic success is the actual moment-to-moment experience and functioning. The Experience Sampling Method (ESM) is a structured diary technique specially developed to appraise subjects in their daily interactions (Delespaul, 1995). The technique makes it possible to study subtle dynamic changes in momentary affective states that are difficult to assess in cross-sectional questionnaires. ESM has high ecological validity (Myin-Germeys et al., 2009; Trull and Ebner-Priemer, 2009) and allows person-tailored data collection. Since it reflects the subject's own daily life, the data facilitates transparent communication and collaborative care. ESM has been used for numerous research purposes in the general population (Jacobs et al., 2013), somatic health care (Parati et al., 2009) and mental health care (Walz et al., 2014). The method proved valuable in monitoring treatment effects (Munsch et al., 2009) and can be used as a treatment intervention (Wichers et al., 2011).

Quality of Life (QoL) is an important outcome indicator. Mental illness often decreases QoL, resulting in lowered subjective well-being and functioning (Fayers and Machin, 2013; Williams et al., 2015). QoL can be defined as “an individual's perception of their position in life in the context of the culture and value systems in which they live and in relation to their goals, expectations, standards and concerns” (The WHOQOL Group, 1995, p. 1405). Researchers and clinicians essentially consider QoL as a subjective concept. A broad operationalization combines different domains, such as social contact, physical health and environmental resources (The WHOQOL Group, 1995; Saxena et al., 1997). Affect, cognition, behavior and physical functioning influence experienced QoL (Spilker, 1990). The World Health Organization Quality of Life Assessment (WHOQOL) was developed to assess this comprehensive, multi-domain view of QoL (The WHOQOL Group, 1995). Other cross-sectional QoL measures either assess the impact of mental illness (Auquier et al., 2003; Fayers and Machin, 2013) or monitor successes in treatment (Ruggeri et al., 2005; Yamauchi et al., 2008; Fleury et al., 2013). Structured interviews are used in the assessment of patients with a severe mental illness, both for subjective information (e.g., self-assessment) and objective information (e.g., social functioning) (Oliver et al., 1997; Priebe et al., 1999). Lehman (1983) showed that patients with severe mental illness were able to give an account of experienced QoL, similar to subjects from the general population.

Barge-Schaapveld et al. (1999) used ESM to study subjective well-being in daily life for patients with depression. They assumed that QoL varies from moment-to-moment and assessed momentary QoL (mQoL) repeatedly, using the question “In general, how is it going with you right now?” ESM questionnaires were administered 10 times a day for six consecutive days. Results confirmed between-subject and within-subject (temporal) variation in mQoL. Compared to healthy control subjects, depressed subjects reported lower mQoL, less activity, and experienced more negative affect and less positive affect. In addition, the variation of mQoL was higher in the depressed group. Furthermore, situational factors had a large influence on mQoL in both groups (Barge-Schaapveld et al., 1999). ESM is a feasible method for the assessment of momentary health-related QoL (Maes et al., 2015). QoL can be measured in the moment, under different daily life situations and varies between and within persons (Barge-Schaapveld et al., 1999; Maes et al., 2015). ESM is a compelling and comprehensive method because it assesses different factors that influence QoL, namely momentary affect and contextual variability.

To date, mQoL was an outcome statistic. Repeated self-assessments are made for a representative sample of moments during a specified period. Individual mQoL assessments are aggregated, yielding a statistic that represents pre or post intervention mQoL for a subject. The clinical relevance is limited, because these aggregated mQoL scores miss the necessary information to inform patients and clinicians on how to improve QoL dynamically over time. This requires an mQoL statistic that directly links treatment aims such as improving well-being with adaptation strategies in the moment and informs individuals and clinicians about choices in daily life.

The mechanism of reward or the process of reward seeking can be relevant to improve mQoL. Subjective well-being is related to reward experiences. Moreover, subjective well-being and reward-related neural activity are related (Gilleen et al., 2015). Reward experiences are the drivers of operant learning (Skinner, 1937). In operant conditioning, people learn from the consequences of their response and use that knowledge to guide future behavior choices (Skinner, 1937; Staddon and Cerutti, 2003). Stimulus-response associations are computed internally and updated frequently, allowing people to predict the outcome and choose responses from their repertoire accordingly. This implicit associative learning is driven by reinforcement. According to behavior theory, reinforcement strengthens or weakens the selection of behavior in a similar situation. Positive reinforcement occurs when a certain response to a new stimulus results in a valuated outcome and is thus rewarded, thereby increasing the likelihood of similar behavior in the future (Flora, 2004).

In mental health research, mechanisms of operant conditioning and reward are repeatedly linked to well-being. Lewinsohn (1974) hypothesized that depression is a consequence of low levels of response-contingent positive reinforcement. In a sample of college students, they correlated depressed mood with time spent on pleasant activities. Increased time in pleasant activities was viewed as an indicator of positive reinforcement. The results showed a moderately negative association: where pleasant activities decreased, experienced depression increased (Lewinsohn and Graf, 1973; Lewinsohn, 1974). A causal link between the two variables could not be established (Sweeney et al., 1982). Other studies have demonstrated positive affective experiences and reward experiences in relation to resilience in depressive subjects (Wichers et al., 2007, 2009). A randomized control trial (RCT) conducted by Geschwind et al. (2011) showed that mindfulness-based cognitive therapy aimed at increasing positive affect and the enjoyment of reward experiences during daily life was associated with a reduction in experienced depression.

Kramer et al. (2014) used an ESM-based intervention (ESM-I) to examine whether self-monitoring of positive affect (PA) is beneficial for depressive patients in addition to treatment as usual. ESM-derived feedback was used as a therapeutic tool to gain insight in implicit dynamic patterns that arise over time. Through person-tailored feedback sessions, hidden patterns were made explicit using visualization in graphs and figures (Kramer et al., 2014). Weekly ESM-I feedback sessions influenced the treatment of depression positively, with an effect still present at 6-month follow-up (Kramer et al., 2014). Contrary to these long-term effects, they found no significant impact of the ESM-I on daily experienced PA during the intervention or shortly after (Hartmann et al., 2015). Another RCT in young adults with depression also provides evidence that ESM-I may have positive impact on pleasure and PA by providing personalized lifestyle advice (van Roekel et al., 2017). Wichers et al. (2015) used ESM as a tool to prospectively observe implicit learning processes for reward-seeking and punishment-avoidant behavior in the context of daily life. They hypothesized that current behavior could be predicted by the experience of related behavior at previous time points. Results confirmed that affect moderates this association over time, both at beep and day level (Wichers et al., 2015).

Experience Sampling Method (ESM) is rooted in ecological psychology where contextual embeddedness is widely recognized (Heft, 2013). Barker introduced the term “behavior settings,” to reflect the mutual relation between human behavior and the environment (Barker, 1965). Behavior settings are eco-behavioral entities which exist independent of persons and form self-regulating systems (Barker, 1968). They represent stable and identifiable constructs with both spatial and temporal indices and can provide opportunities or constrain the actions of persons (Barker, 1965; Wicker, 2012). The term behavior setting puts emphasis on processes and structures that often go unnoticed in the daily life of individuals (Wicker, 2012).

Imagine yourself on a market. Being there with friends will likely provide a different experience than being alone. In addition, activities influence the experience. You could be working, buying a last ingredient for dinner or simply enjoying yourself. Other factors play a role as well, such as the location of the market, its attributes, the weather and time of day. All ingredients form the behavior setting—the rich and meaningful context.

Future research should emphasize the linkage between a person's affective experience and behavior setting characteristics, including the beliefs and know-how of this behavior setting (Wicker, 2012). With ESM, information on momentary affect can be gathered in the context of daily life (Delespaul, 1995). Behavior settings are important because they provide insight in the contextual variability of positive or negative affect. Clinicians can coach patients to engage in contexts (i.e., future behavior selection) to maximize individual patterns of positive affect and avoid negative affect, leading to more experienced QoL.

In line with the literature above, subjects can improve QoL by engaging more often and for longer time in affectively rewarding situations. Some may argue that extremely rewarding, low frequency behaviors can boost QoL (e.g., a holiday travel compensates for a boring job). However, exceptional situations do not rule moment-to-moment experiences, while frequent minor events that occur naturally in the flow of daily life have an impact on mental well-being (Peeters et al., 2003). The pursuit of reward cannot be a unidimensional focus. QoL does not increase linearly (more is better), but optimizes by balancing challenges of daily life. Even the most enjoyable job will be perceived differently when there is no time to relax anymore. Maximizing reward experiences in daily life, means spending most of our time in rewarding situations (eliciting high PA) and avoiding situations with low PA. Depressive patients, for instance, are out of balance and spend insufficient time on pleasant activities (Lewinsohn, 1974; Peeters et al., 2003; Thompson et al., 2012; Roekel et al., 2016). Their time budgets are not optimized and too much time is spent in behavior settings with low PA.

The reward-related QoL function (rQoL) reflects the momentary fit between context and optimal affective experience. ESM can monitor this process in treatment (Kramer et al., 2014). rQoL can be used in shared decision-making. Feeling good in some situations and bad in others is a transparent communication and most patients understand this intuitively. The collaborative ESM feedback sessions between patients and clinicians are the setting to discuss reward optimization. In a shared decision process, patients and clinicians select rewarding situations and explore how to increase the occurrence of well-being in daily life.

Can these situations be detected and personal profiles computed? When applied in clinical practice, does this improve the subject's overall well-being? This paper describes the development of a reward-related QoL function (rQoL) and the proof of concept of its applicability. A reward statistic is defined. It reflects individual daily life moment-to-moment variation in reward efficiency, by combining the actual (positive) affect with the occurrence frequency of the actual behavior setting. Data is collected using ESM. To our knowledge, this is the first study that uses mechanisms of reward to design a momentary quality of life measure.

## Method

### Sample

To assess the feasibility of the rQoL concept, ESM data from an existing dataset–the D-STIGMI study (van Zelst, 2014 p. 103) was used. The D-STIGMI study evaluates a psycho-educational coping skill training in people with severe mental illness using a RCT. The aim of the training was to increase resilience against stigmatization. The ESM data collection was an optional add-on to explore innovative outcome parameters. The Medical Ethics Committee of Maastricht University Medical Centre approved the study protocol under the number of NL3179406810. In the current study, only baseline ESM assessment data were used as seeds for the random simulated sets. ESM data were available for 27 participants.

### Measurements

#### Experience sampling method

Experience Sampling Method (ESM) is a structured diary technique to assess moment-to-moment mental state changes in relation to situations in the daily life of individuals (Delespaul, 1995; Jacobs et al., 2011). The data of the D-STIGMI study was collected with the PsyMate™ device, a palm-top assessment tool developed for ESM data collection (http://www.psymate.eu/)^1^. The PsyMate™ was programmed to emit 10 beeps each day for six consecutive days. Beeps were generated at semi-random moments, within 90-min blocks, between 7.30 and 22.30 h. An auditory and visual signal indicated the availability of a short questionnaire. Responding lasted less than a minute. The questionnaires remained available for 15 min and subjects were instructed to promptly reply. Daily life experiences are captured in items assessing current affect, activities and context. Most items were rated on a 7-point Likert scale (ranging from 1 = “not at all” to 7 = “very”). A bipolar scale (−3 = negative, 0 = neutral, to 3 = positive) was used to assess stressful events. Context (activity, location and person present) and the use of substances were assessed with multi-optional checklists. The item “Overall, I feel well at the moment” was added to assess mQoL. Beeps are considered valid when the whole questionnaire is completed. Each subject could respond to a maximum of 60 beeps. In line with ESM guidelines, subjects were included in the analyses when they completed at least a third of the beeps (20 valid beeps) (Delespaul, 1995).

### Assessment of rQoL

Momentary reward-related quality of life (rQoL) is defined as the fit between frequency of situations (behavior setting) and actual mental state. rQoL can be computed for each moment and provides feedback to clinicians and patients to collaboratively select intervention strategies that optimize the mental state in daily life and lead to more overall well-being. The related rQoL statistic uses the subject's own data to assess if he/she optimizes the selection of contexts to maximize positive mental states, meaning that rewarding situations should occur often and less rewarding situations should be avoided.

#### The ingredients of the function

##### Mental states

The ESM questionnaire contains mood items that assess PA: I feel “cheerful,” “satisfied,” “relaxed,” and “enthusiastic.” Momentary PA was normalized by subject [*z*PA_(ij)_ = PA_(ij)_ − PA_(i)_-with “i” for subjects and “j” for moments]. *z*PA_(ij)_ yields positive scores for better than average mental states and negative scores for below average mental states. Better than average mental states are assumed to be rewarding.

##### Behavior setting

A meaningful situation is a behavior setting. Time, place, persons and activities define them. The ESM definition of a behavior setting uses the context information available in the beep-level questionnaires: the time of the day (morning, afternoon, evening), persons present (“with whom am I”: “partner,” “resident family,” “family living away from home,” “friends,” “colleagues,” “acquaintances,” “strangers/others,” and “nobody”), activity (“what am I doing”: “resting,” “work/school,” “household/groceries,” “hygiene,” “eating/drinking,” “relaxation,” “doing something else” and “nothing”) and location (“where am I”: “home,” “someone else's home,” “work/school,” “public place,” “on the go,” and “somewhere else”). This results in 3 × 8 × 8 × 6 = 1,152 potential combinations, of which many infrequently occur or never occur and result in empty cells for individual subjects. The detailed number of situations does not allow the selection of high and low frequency behavior settings for each subject. Therefore, the time of the day was omitted and the number of options for who, what and where limited to six each. For persons present, “partner” was included into “resident family” and “colleagues” into the “acquaintances” category. For activity, we combined “resting” with “doing nothing” and “household/groceries” with “hygiene.” The options for location remained unchanged. The occurrence (as a proportion) of the 216 resulting behavior settings was computed for each individual subject. The cumulative proportion was computed with a break at 0.5 to differentiate the large set of infrequent situations and the much smaller set of frequent situations.

##### Reward function

The momentary rQoL statistic combines the frequency of the momentary behavior setting with the actual mental state. Specifically, the function uses the normalized positive affect score by individual [*z*PA_(ij)_] and weights it with the frequency of occurrence of the individuals' behavior setting [cp(BS_ij_)_i_]. Reward efficiency occurs when high frequency situations yield positive moods. High rQoL occurs when high frequency situations are combined with positive mental states or low frequency is limited to negative mental states. Low rQoL situations combine poor mental states with high frequency situations or elevated mental states with infrequent behavior settings. These characteristics are reflected in the formula:

$$\begin{array}{l} rQoL{\left(PA\right)}_{ij}=zPA_{ij}\times \left({cp\left(BS_{ij}\right)}_{i}-0.5\right) \end{array}$$

in which:

*rQoL(PA)*_*ij*_ is reward-based *QoL* computed on *PA* for subject i on moment *j*; *zPA*_*ij*_ is the standardized *PA* score for subject *i* at moment *j*; and *cp(BS*_*ij*)*i*_ is the cumulative proportion for subject *i* of occurrence of the current behavior setting for that subject *i* on moment *j*.

−0.5 is used to generate a cut-off score for high and low proportions. Other cut-off scores were explored (and are further explained in the analyses section).

Using this formula, a specific rQoL score was generated for each ESM moment. Negative scores (e.g., −0.1) represent low rQoL, whereas positive scores (e.g., 0.9) represent high rQoL. An alternative reward function can be defined with negative affect (NA) but PA was selected in line with other scholars in the field (Wichers et al., 2009, 2015). The rQoL statistic was computed using a small program written in a Stata™ (v13.0) script.

### Analyses

To assess the feasibility of the rQoL function, we assessed the impact of different choices using resampling methods (Monte Carlo experiments) to generate ESM data for virtual subjects. The sample size (*N*) is the number of ESM observations or beeps drawn from D-STIGMI dataset used as seeds. Different selections of contextual domains and different cut-off scores were explored to generate rQoL. Simulations were run using virtual subjects with data extracted from all beeps from the D-STIGMI seeding database. Because real subjects have more specific frequency distributions in behavior settings, virtual datasets were extracted for subjects with low, average and high situational diversity.

#### Which sample size do we need to reliably assess behavior setting in individuals?

A Monte Carlo experiment was executed to study the effect of the number of available beeps on the average number of unique context combinations (behavior settings). Three different definitions for behavior setting were explored: a What only definition (BS_W: 6 situations), a What and Who–based definition (BS_WW: 6 × 6 = 36 situations) and a What, Who and Where definition (BS_WWW: 6 × 6 × 6 = 216 combinations). Resampling made it possible to explore the alternative behavior setting definitions for different sampling sizes (20, 40, 60, 80, 100, 250, 500, 750, 1,000, 2,000, 3,000, 4,000, 8,000, 10,000 “valid” observations by virtual subject).

#### Which sample size do we need for optimal variation in reward (rQoL)?

A second Monte Carlo experiment was executed to study the effect of available observations on the average scores and variation of rQoL, independently for BS_W, BS_WW and BS_WWW. This was done to provide insight in the number of observations needed to reliably generate rQoL. Initially, different cut-off scores (0.50, 0.40, 0.30, and 0.20) were explored to differentiate low and high frequency situations. Only the cut-off score 0.50 proved sensitive enough for meaningful rQoL detection and was therefore used in further analyses. Sample sizes that were explored, are 20, 40, 60, 80, 100, 250, 500, 750, 1,000, 2,000, 3,000, 4,000, 8,000, and 10,000 observations for the virtual subjects in the simulation.

#### What is the effect of the actual contextual variation in real subjects?

In contrast to virtual subjects who live in the contexts of the group of individuals, actual subjects live in environments that are more restricted. We selected subjects with different levels of behavior setting differentiation (the number of non-empty BS_WWW categories) and ranked these individuals to compute percentiles. A Monte Carlo experiment was executed to simulate the rQoL functions for subjects with low (5th percentile), average (50th percentile) and high (95th percentile) variability in contextual domains. Sample sizes that were explored are 20, 40, 60, 80, and 100 “valid” observations by virtual subject.

#### Is reward-related rQoL something different than positive affect?

A final Monte Carlo experiment was executed to assess whether momentary PA and rQoL were separate concepts. Pearson's product-moment correlations between PA scores and rQoL scores were computed for each resampled set of momentary data, using varying sampling sizes. This was done separately for BS_WWW, BS_WW and BS_W in the overall sample.

The seeding data for the Monte Carlo experiments were selected using ESM observations from real subjects combined together as the sampling domain. When not enough unique empirical data were available (simulated samples exceeding available observations), we sampled with replacement. For each simulation, 1000 samples were drawn. Analyses were performed in Stata™ (v13.0). The do-file is added in the Supplementary Material.

## Results

### Subject characteristics

Twenty-seven patients with a severe mental illness were included in the ESM baseline measurement of the D-STIGMI study. Four patients had insufficient valid beeps (< 1/3), thus 23 patients were included as seeds for Monte Carlo experiments. The sampling set includes 1058 valid beeps, at average 48 per subject (*SD* = 9.03, range 22–63). No significant differences were found between the original sample and the seed-sample on age (*p* = 0.97) and sex (*p* = 0.53). Demographic information is summarized in Table 1.

**Table 1:** Demographics and characteristics of the 23 participants seed-sample.

**Characteristics**	***N* (%)**	**Mean**	***SD***	**Range**	**Details**
Age (years)		46	9.95	29-62	
**GENDER**					
Female	60				
Male	40				
**DSM-IV DIAGNOSTICS**					
Single diagnosis	61				
Multiple diagnoses	35				
Missing diagnostics	4				
**CLINICAL CLASSIFICATION**					
Primary psychotic vulnerability	68				(292.12/295.30/295.70/298.9/296.40)
Primary non-psychotic vulnerability	32				(296.31/299.80/301.83/301.83/309.81)
GAF symptoms		46.45	14.27	28–70	
GAF handicap		45.00	9.83	31–70	
**BEEP CHARACTERISTICS (*****N*** = **1,058)**					
Positive affect (overall beeps)		4.00	1.41	1–7	
Positive affect (by subject)		4.00	0.99	1.3–6.3	
**NUMBER OF CONTEXT COMBINATIONS**					
Unique BS_WWW combinations	113				Possible = 216
Unique BS_WW combinations	36				Possible = 36
Unique BS_W combinations	6				Possible = 6
**CONTEXT COMBINATIONS PER SUBJECT**					
Low BS variability subject	5				
Average BS variability subject	17				
High BS variability subject	28				

*292.12 = Induced psychotic disorder, with hallucinations. 295.3 = Schizophrenia: Paranoid type. 295.7 = Schizoaffective disorder. 298.9 = Psychotic disorder NOS. 296.4 = Bipolar I disorder, most recent episode hypomanic. 296.31 = Major depressive disorder, mild. 299.8 = Rett's disorder; Asperger's disorder; Pervasive developmental disorder NOS. 301.83 = Borderline personality disorder. 309.81 = posttraumatic stress disorder*.

*GAF, Global Assessment of Functioning; BS, Behavior Setting*.

### Which sample size do we need to assess behavior settings in individuals reliably?

**Figure 1:**
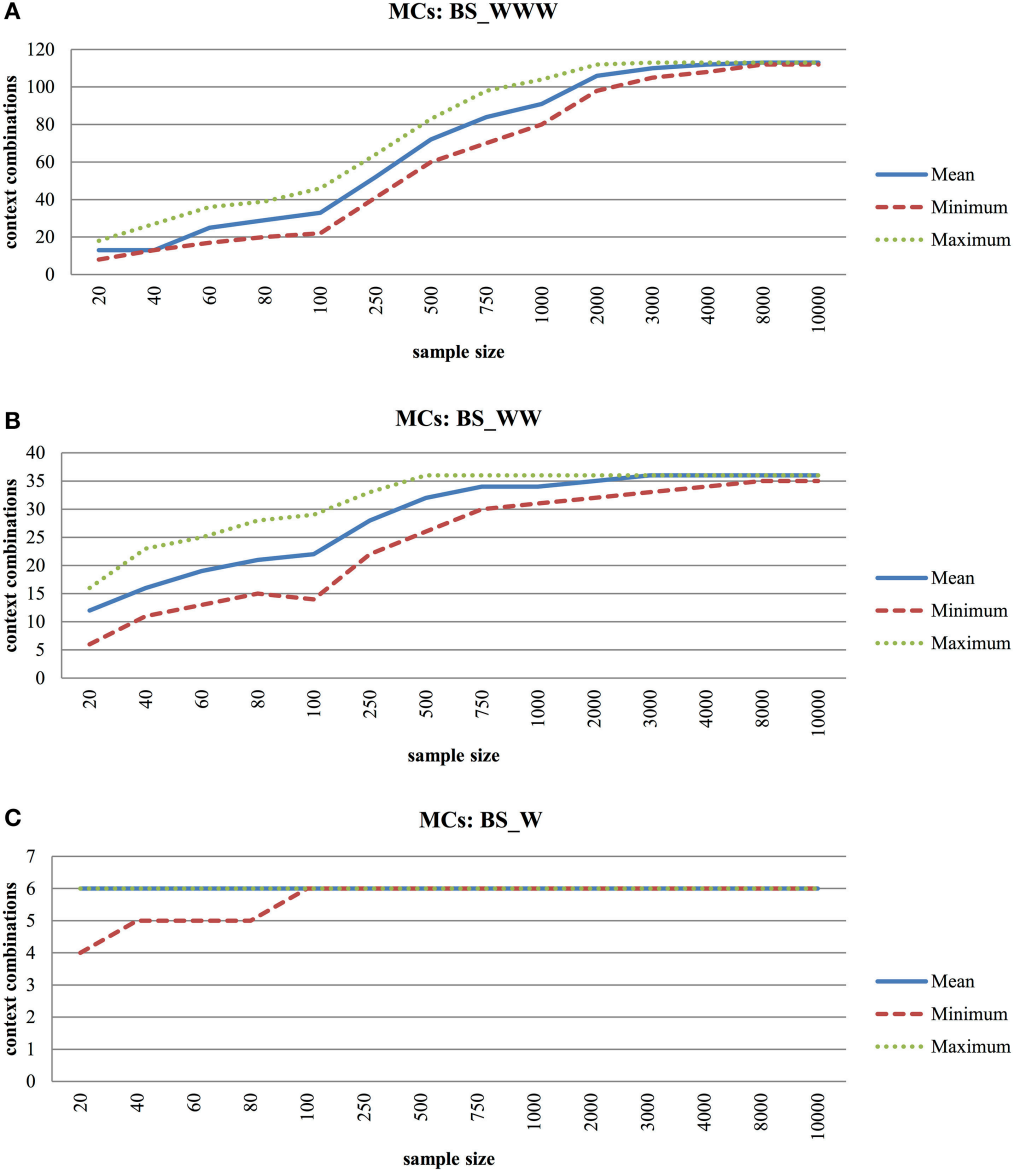
**(A)** Monte Carlo Experiment (MCs) to Explore Average-, Minimum-, and Maximum Number of Unique Context Combinations with Increased Sample Size for Behavior Settings Including Who, What, Where Information (BS_WWW). **(B)** Monte Carlo Experiment (MCs) to Explore Average-, Minimum- and Maximum Number of Unique Context Combinations with Increased Sample Size for Behavior Settings Including Who, What Information (BS_WW). **(C)** Monte Carlo Experiment (MCs) to Explore Average-, Minimum- and Maximum Number of Unique Context Combinations with Increased Sample Size for Behavior Settings Including What Information (BS_W).

Figure 1 shows the number of unique behavior settings generated by the resampling procedures for different sample sizes. The theoretical number of behavior settings was 216 but the beeps actually only contained 113 options (the empirical ceiling). The graph has three phases: from *N* = 20 (*mean* = 13; *SD* = 1.75) to *N* = 100 (*mean* = 33; *SD* = 3.31), from *N* = 100 to *N* = 1,000 (*mean* = 92; *SD* = 3.6) and from *N* = 1,000 to *N* = 10,000 (*mean* = 113; *SD* = 0.16). Around *N* = 8,000 (*mean* = 113; *SD* = 0.16), saturation is reached. Standard errors are low in small samples (*SEM* = 0.39; *N* = 20) and increase over the second phase (max *SEM* = 4.08; *N* = 500) and finally to reduce again (*SEM* = 0.002; *N* = 10,000). Figures 1B,C reflect simulations for the BS_WW (theoretical 6 × 6 = 36 options, sample 36 options) and BS_W behavior settings (theoretical 6 options, sample 6 options). The same pattern replicates but the ceiling is reached with smaller samples (around 500 observations in the BS_WW and 100 in the BS_W situation).

### Which sample size do we need for optimal variation in reward quality of life (rQoL)?

**Figure 2:**
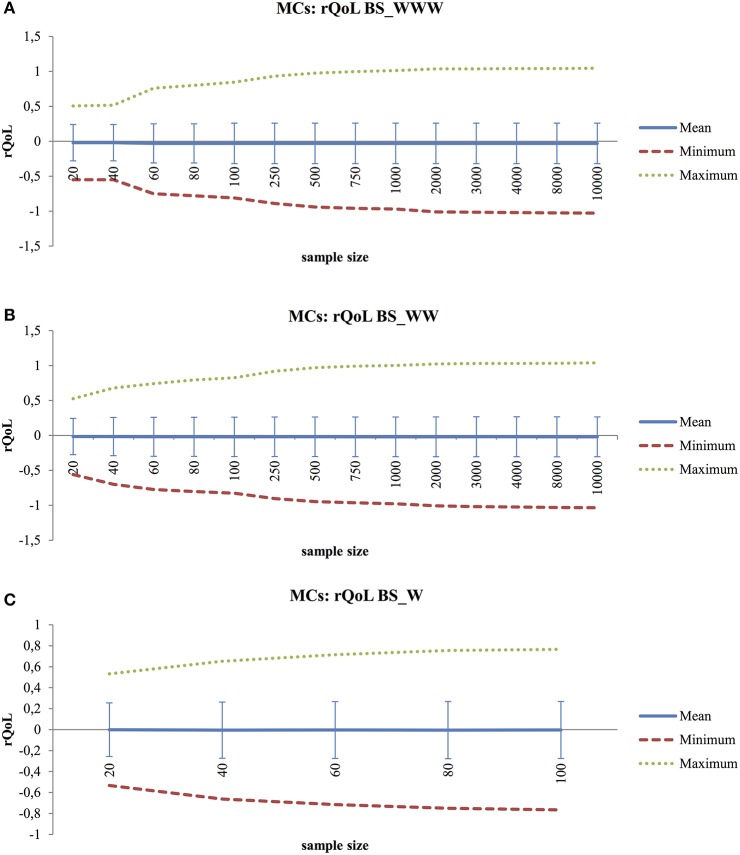
**(A)** Monte Carlo Experiment (MCs) to Explore Average-, Minimum-, Maximum- and Standard Deviation Scores of rQoL(PA) with Increased Sample Size for Behavior Settings Including Who, What, Where Information (BS_WWW). **(B)** Monte Carlo Experiment (MCs) to Explore Average-, Minimum-, Maximum-, and Standard Deviation Scores of rQoL(PA) with Increased Sample Size for Behavior Settings Including Who, What Information (BS_WW). **(C)** Monte Carlo Experiment (MCs) to Explore Average-, Minimum-, Maximum-, and Standard Deviation Scores of rQoL(PA) with Increased Sample Size for Behavior Settings Including What Information (BS_W).

For different sampling sizes, the rQoL was computed at each moment of the simulated virtual subject. As expected, all samples had an average of 0.00, the neutral point of the rQoL function. Standard errors of the mean were low and are considered negligible (*SEM* = 0.06 to *SEM* = 0.003). For BS_WWW and BS_WW (Figures 2A,B), the range of rQoL scores increased up to sample sizes of 500 beeps. For BS_W the range was more restricted but reached its maximum for sampling sizes of 60.

### What is the effect of the actual contextual variation in real subjects?

**Figure 3:**
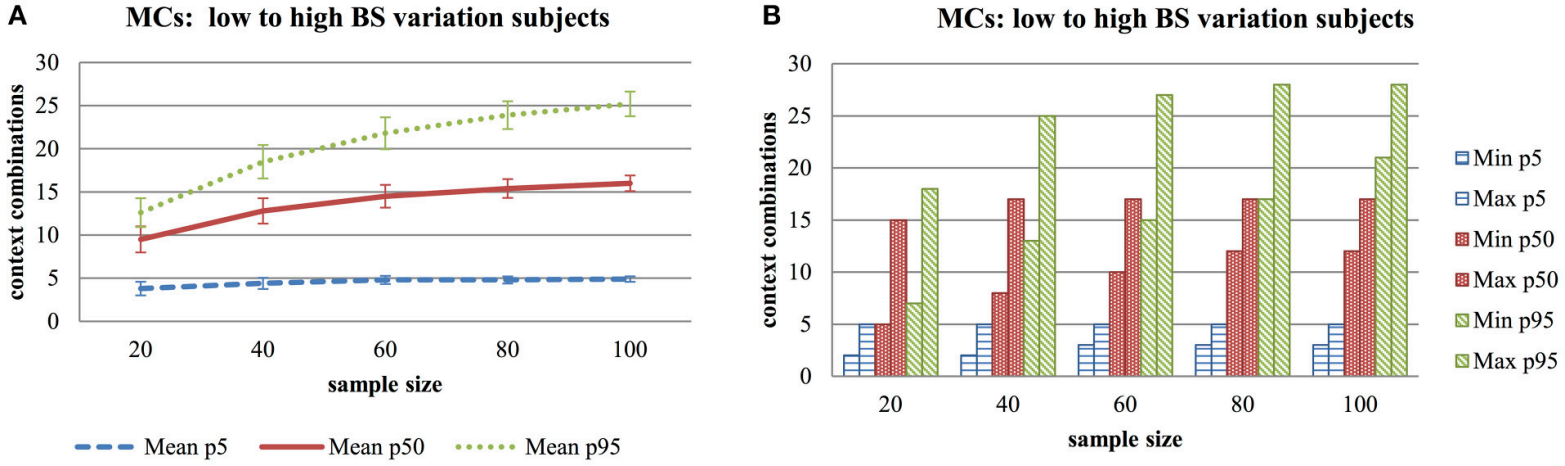
**(A)** Monte Carlo Experiment (MCs) to Explore Average- and Standard Deviations Scores of Unique Context Combinations for Subjects with Low-, Medium- and High Diversity in Behavioral Setting, with Increased Sample Size for Behavior Settings Including Who, What, Where Information (BS_WWW). **(B)** Monte Carlo Experiment (MCs) to Explore Minimum- and Maximum Number of Unique Context Combinations for Subjects with Low-, Medium- and High Diversity in Behavioral Setting, with Increased Sample Size for Behavior Settings Including Who, What, Where Information (BS_WWW).

The selected subjects (low-, average-, high variability in behavior setting) responded reliably to respectively 31, 45, and 55 beeps, with respectively 5, 17, and 28 different behavior settings (using the BS_WWW combination). Results are presented in Figure 3. Part 3a shows the increase in average rQoL scores for the subject with low behavior variability (p5; 5th percentile), average behavior setting variability (p50; 50th percentile) and high behavior setting variability (p95; 95th percentile). Increases in sampling size did not affect the scores for subjects with low behavior variation (1.1 mean difference), but did for subjects with high variation (12.6 mean difference). Part 3b shows the range of minimum and maximum scores. This confirms the previous observation. Smaller sampling sizes are possible in subjects living restricted lives.

### Is reward-related QoL something different than positive affect?

**Figure 4:**
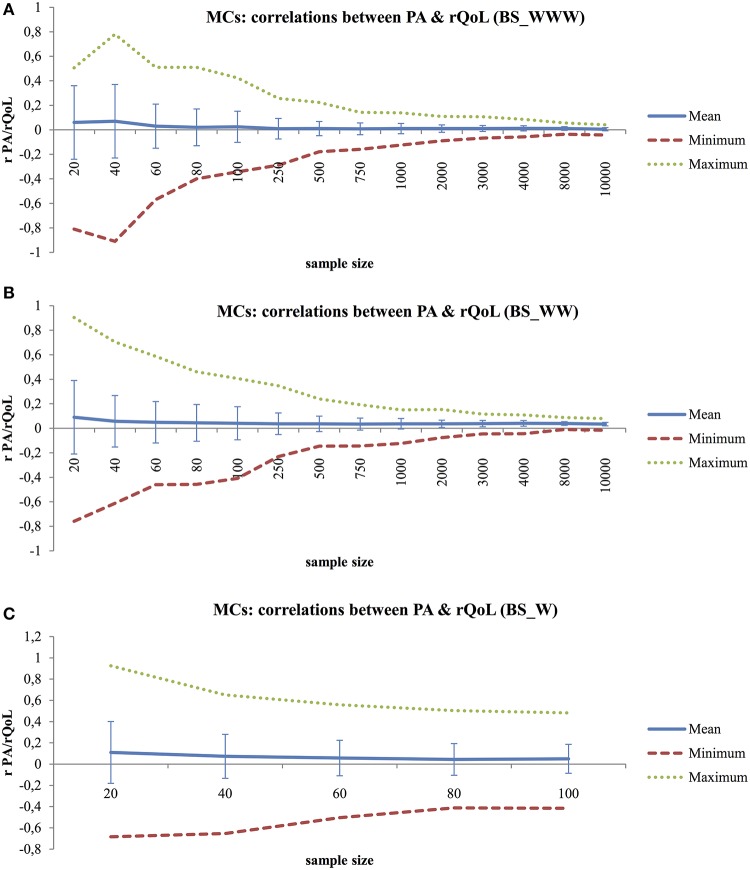
**(A)** Monte Carlo Experiment (MCs) to Explore the Relationship between PA Scores and rQoL Scores with Increased Sample Size for Behavior Settings Including Who, What, Where Information (BS_WWW), Using Pearson's Product-Moment Correlations. **(B)** Monte Carlo Experiment (MCs) to Explore the Relationship between PA Scores and rQoL Scores with Increased Sample Size for Behavior Settings Including Who, What Information (BS_WW), Using Pearson's Product-Moment Correlations. **(C)** Monte Carlo Experiment (MCs) to Explore the Relationship between PA Scores and rQoL Scores with Increased Sample Size for Behavior Settings Including What Information (BS_W), Using Pearson's Product-Moment Correlations.

The Pearson's product-moment correlations of the Monte Carlo experiments on sampled sets of PA scores and rQoL are summarized in Figure 4. Looking at the average correlation scores, weak associations were found between PA and rQoL for BS_WWW, BS_WW, and BS_W [range *r*_(18)_ = 0.11, *p* < 0.001 to *r*_(9, 998)_ = 0.005, *p* = < 0.001]. The range between minimum and maximum correlational scores in all three variations of behavior setting is large in smaller sample sizes (highest difference from *min* = −0.91 to *max* = 0.90) and decreases with larger sample sizes (lowest difference from *min* = −0.02 to *max* = 0.04).

## Discussion

### General conclusion

The purpose of the current proof-of-concept study is two-fold. First, a momentary rQoL statistic was defined. Second, Monte Carlo experiments were performed with samples of beep-level data for virtual subjects to check the feasibility and initial validity of the statistic. Momentary rQoL integrates affective experience (positive affect) and situations (behavior setting). A cut-off score of 0.50 of the cumulative proportion was chosen to separate low and high frequency situations. This proved to be the best choice to detect relevant contexts for reward efficiency in most subjects. Results show that the rQoL statistic is feasible. The rQoL statistic is defined at the moment level, allowing assessment of change over time. At a specific moment in time, a positive rQoL score indicates good reward efficiency, whereas a negative score indicates bad reward efficiency.

Persons present (Who), activity (What) and location (Where) were used to define different conceptualizations of a behavior setting, namely Who-What-Where (BS_WWW), Who-What (BS_WW), and What (BS_W). For Who-What-Where combinations, not all theoretical possibilities were available in the reference beep dataset. This dataset included 113 unique combinations out of the 216 theoretical options. Some situations had low frequencies (e.g. working with your partner or doing household/groceries at work) or simply did not occur in the group of patients with a severe mental illness. First, Monte Carlo experiments were performed to check which sample size is needed for reliable behavior setting calculations in individuals. Overall, large sample sizes were needed to generate realistic frequency distributions (time budgets) in virtual subjects (saturation was reached at *N* = 8,000 for BS_WWW, *N* = 500 for BS_WW, and *N* = 100 for BS_W). Next, Monte Carlo experiments were performed to explore which sample size is needed to detect optimal rQoL variation. The range of rQoL scores increased up to generated samples of 500 beeps for BS_WWW and BS_WW, whereas the limit was reached at 60 beeps for BS_W. Therefore, a behavior setting defined by Who-What proved most useful: it provides sufficient behavior setting variation to generate a reliable frequency distribution at acceptable sample sizes. An optimal spread in rQoL variation is obtained after collecting 500 observations, meaning that all possible behavior-setting combinations are present in the sampling period (see Figure 2B). However, a minimum of 100 beeps is required to reliably calculate the rQoL statistic. The sample sizes needed for BS_WWW are unrealistic in ESM. These results suggest that an extended sampling period is needed before the rQoL statistic can be integrated as an active part of treatment.

Further, Monte Carlo experiments were used to explore actual situational variations in real subjects with low, average and high behavior setting variation with combinations of persons present, activity and location as behavior setting. This behavior setting definition was chosen because it theoretically provides the largest chance of finding a decent spread in unique situations in this patient group. Results show that small sample sizes are possible in subjects with low behavior setting variations (*N* > 40), whereas larger sample sizes (*N* > 100) are needed for subjects with average and high behavior setting variations. It is difficult to assess which definition of behavior setting is sufficient for individual subjects. For now, behavior settings defined by Who-What-Where combinations seem the best option because the restricted living environments of individual subjects results in less overall situational variation.

Finally, Monte Carlo experiments were run to see whether momentary rQoL is distinct from PA. Only weak correlations were found and results confirmed that the concepts assess different aspects of daily life mental states. Additionally, the momentary rQoL scores were correlated with “In general, how is it going with you right now” (mQoL). For this, the overall beep sample of 1058 valid observations was used. Results show a moderate positive correlation [*r*_(1, 056)_ = 0.33, *p* < 0.001], with mQoL explaining 11 percent of the variation in rQoL.

### Strengths

To our knowledge, this is the first study that combines affective experience with behavior settings in an operationalization of rQoL in daily life. A main strength is the use of ESM data collected in the flow of daily life, making the rQoL statistic highly specific to the situation of the subject. Monte Carlo experiments are especially suited for exploration of properties and sampling characteristics of specific combined statistics (Mooney, 1997). Researchers compute different parameters, but insufficiently realize the relation and biases due to sampling characteristics. Knowing how a statistic responds to different sampling characteristics is particularly important when applied in treatment.

## Limitations

The choices made to operationalize momentary rQoL can seem arbitrary. Other options could be explored. For example, the rQoL statistic can be computed with negative affect. Additional cut-off scores could be explored to differentiate low and high frequency situations. The behavior settings are defined on persons present, activity and location, although more aspects of the environment could be relevant. Behavior settings are complex entities which include a number of contextual variables (Barker, 1965). Here we excluded, for example, temporal indices such as the time of the day and limited the categorical options so that a critical mass of workable data remains. With advances in technology, other factors such as heart rate or weather reports could be more easily combined with ESM data, thereby increasing the accuracy of the behavior setting. In the future, it is possible to harvest big data, such as GPS location, sensor data, or geo-political events to enrich the situational information without increasing subject burden. However, the main purpose of this study was to explore a first operationalization based on available ESM data and test the behavior of the momentary rQoL statistic using Monte Carlo experiments.

Furthermore, the generated samples using real subject data (for the analysis of subjects with low-, average- and high behavior setting variability) were oversaturated and the same records were used repeatedly (due to replacement). The used sample included insufficient subjects with large beep datasets (>1000 observations). It would be interesting to replicate these analyses when longer series become available.

Another limitation is the use of a specific sample of patients with severe mental illness as seeds for the Monte Carlo experiments. These subjects often lead restricted lives with limited variation in daily life activities (Lewinsohn, 1974; Holloway and Carson, 2002). It would be interesting to see how the frequencies in behavior setting differ between these patients and the general population. This first operationalization was made from a clinical perspective, to explore the possibility of an rQoL statistic that is meaningful to patients with severe mental illness. However, the basic components of the rQoL statistic could be relevant across clinical populations. For example, patients with depression or anxiety could also use the statistic within treatment to optimize their balance in rQoL. Future research should use Monte Carlo simulations on ESM data collected in other populations; to see how the frequency in behavior setting is distributed, to calculate which sample size would work best, and to see in what situation the rQoL statistic can be meaningfully calculated. It is conceivable that situational variability differs between populations (and between individuals).

### Implications and further research

This proof-of-concept study indicates that momentary rQoL is a feasible statistic. Monte Carlo experiments provide valuable insight in the behavior of the statistic under different sampling restraints. The methodology can be used to further improve rQoL. Monte Carlo experiments should be used more frequently in ESM studies. Several suggestions were made for future research. The question remains whether reward-related optimized well-being is actually quality of life; maybe a better description is more adequate. The link between the rQoL statistic and mQoL could further be explored, as well as the relation of rQoL with other (cross-sectional) measures of QoL. Furthermore, the statistic should be explored in other populations that engage in more diversified behavior settings.

It is interesting to explore whether the rQoL statistic improves targeted communication with patients. The statistic could be used to identify situations that result in low rQoL, so that changes can be made in daily life and progress can be monitored (see the Supplementary Material for a hypothetical case example). A previous ESM-based feedback intervention (Kramer et al., 2014) improved therapeutic outcome. The question remains if well-being can be improved by a person-tailored rQoL feedback intervention that monitors reward experiences in daily life. Shared decision-making is facilitated when clinicians and patients share the same information. ESM data, disclosed by smart feedback, can provide this context. By integrating the clinician's expertise with the goals and knowledge of patients and relatives, and by looking at environmental daily life challenges and opportunities, more suggestions that are realistic can be made for optimizing reward in daily life, possibly leading to improved well-being and QoL.

## Author contributions

SV and PD worked on the conceptualization of rQoL and subsequent analyses. SV wrote the initial article. All authors (SV, PD, CS, and CvZ) provided substantial feedback to the rationale of the article and contributed to later versions of the article.

### Conflict of interest statement

The authors declare that the research was conducted in the absence of any commercial or financial relationships that could be construed as a potential conflict of interest.

## Footnotes

^1^Internet site: www.psymate.eu
