# Supplementary material for: Constructing a Reward-Related Quality of Life Statistic in Daily Life—a Proof of Concept Study Using Positive Affect
Source: Front Psychol. 2017 Nov 2;8:1917. doi: 10.3389/fpsyg.2017.01917 (PMC5673660; doi:10.3389/fpsyg.2017.01917)

Supplementary Material

Constructing a Reward-Related Quality of Life Statistic in Daily Life

# – a Proof of Concept Study Using Positive Affect-

Simone J. W. Verhagen[1*], Claudia J. P. Simons[1,2], Catherine van Zelst[1,] Philippe A. E. G. Delespaul[1,3]

*** Correspondence:** Simone Verhagen: simone.verhagen@maastrichtuniversity.nl

List of abbreviations

ESM	Experience Sampling Method (or EMA: Ecological Momentary Assessment)
rQoL	reward-related Quality of Life
mQoL	momentary Quality of Life
MCs	Monte Carlo simulations
BS	Behavior Setting (context or situation)
BS_W	BS: What = situation defined by current activity
BS_WW	BS: What Who = situation defined by combining current activity and persons present
BS_WWW	BS: What Who Where = situation defined by combining current activity, persons present and location
PA	Positive Affect
NA	Negative Affect
N	Number of observations or so-called beeps

Clinical implications rQoL statistic –hypothetical case example-

Patient X has used ESM for several weeks to monitor her progress towards recovery. She struggles with her daily routine and does not feel satisfied with the way her life is going at the moment. However, she does not really know what to change in her life to feel satisfied again. Together with her clinician, ESM data are discussed using an online reporting page that explores the subject's mental state and context using interactive graphs and figures. The reporting page is intended as a tool for collaboration and can be used to generate functional analyses. For each beep moment, a rQoL score is presented on the screen, ranging from negative to positive, with 0 as a neutral point. A positive score represents feeling good in a situation that also occurs frequently (plus-plus) or feeling less good in a situation that is infrequent (minus-minus). Negative scores reflect an imbalance in daily life reward experiences (feeling good in infrequent (plus-minus) or bad in frequent situations (minus-plus). Series of rQoL scores are displayed over time, to identify time trends and outliers. At each time point, the rQoL score can be explored to zoom into the behavior setting of that moment. The exploration assesses both strengths and weaknesses of the individual. Together with the clinician, patient X decides on action points that could lead to small changes in daily life that are deemed realistic. Continued monitoring provides the necessary feedback to see if progress can be made towards improved reward-efficiency and ultimate to well-being.

STATA™ (v13.0) do file for Monte Carlo experiments

```
*ssc install egenmore

clear all

program define mc_reward, rclass
file open Total_OK using "C:\...\result_file_of simulations.csv", write replace

* writing header

	file write Total_OK "Iteration"
	file write Total_OK _tab
	file write Total_OK "cnt catheg"
	file write Total_OK _tab
	file write Total_OK "Mean rPA"
	file write Total_OK _tab
	file write Total_OK "sd rPA"
	file write Total_OK _tab
	file write Total_OK "min rPA"
	file write Total_OK _tab
	file write Total_OK "max rPA"
	file write Total_OK _tab
	file write Total_OK "corr PArPA"
	file write Total_OK  _n

set more off

* iterations

forvalues xi=1(1)1000 {
	clear
	use "C:\...\reward_datafile_of_ESM_seeds.dta"
	bsample 100
	egen cnt_bs=nvals(behav_setting)
	egen mPA=mean(sub_pa)
	egen sdPA=sd(sub_pa)
	gen zPA=(sub_pa-mPA)/sdPA
	so behav_setting
	by behav_setting:egen cfreq=count(behav_setting)
	so cfreq behav_setting
	egen crfreq=rank(cfreq)
	generate crprop=crfreq/_N
	gen rPA=zPA*(crprop-.50)
	summarize cnt_bs
	file write Total_OK "Iteration"
	file write Total_OK (`xi')
```

```
        file write Total_OK _tab
        file write Total_OK (string(r(mean)))
        summarize rPA
        file write Total_OK _tab
        file write Total_OK (string(r(mean)))
        file write Total_OK _tab
        file write Total_OK (string(r(sd)))
        file write Total_OK _tab
        file write Total_OK (string(r(min)))
        file write Total_OK _tab
        file write Total_OK (string(r(max)))
        correlate sub_pa rPA
        file write Total_OK _tab
        file write Total_OK (string(r(rho)))
        file write Total_OK  _n
}

file close Total_OK
clear
import delimited "C:\...\result_file_of simulations.csv"
su
histogram cnt catheg
histogram mean rpa
```

Overview Monte Carlo experiments

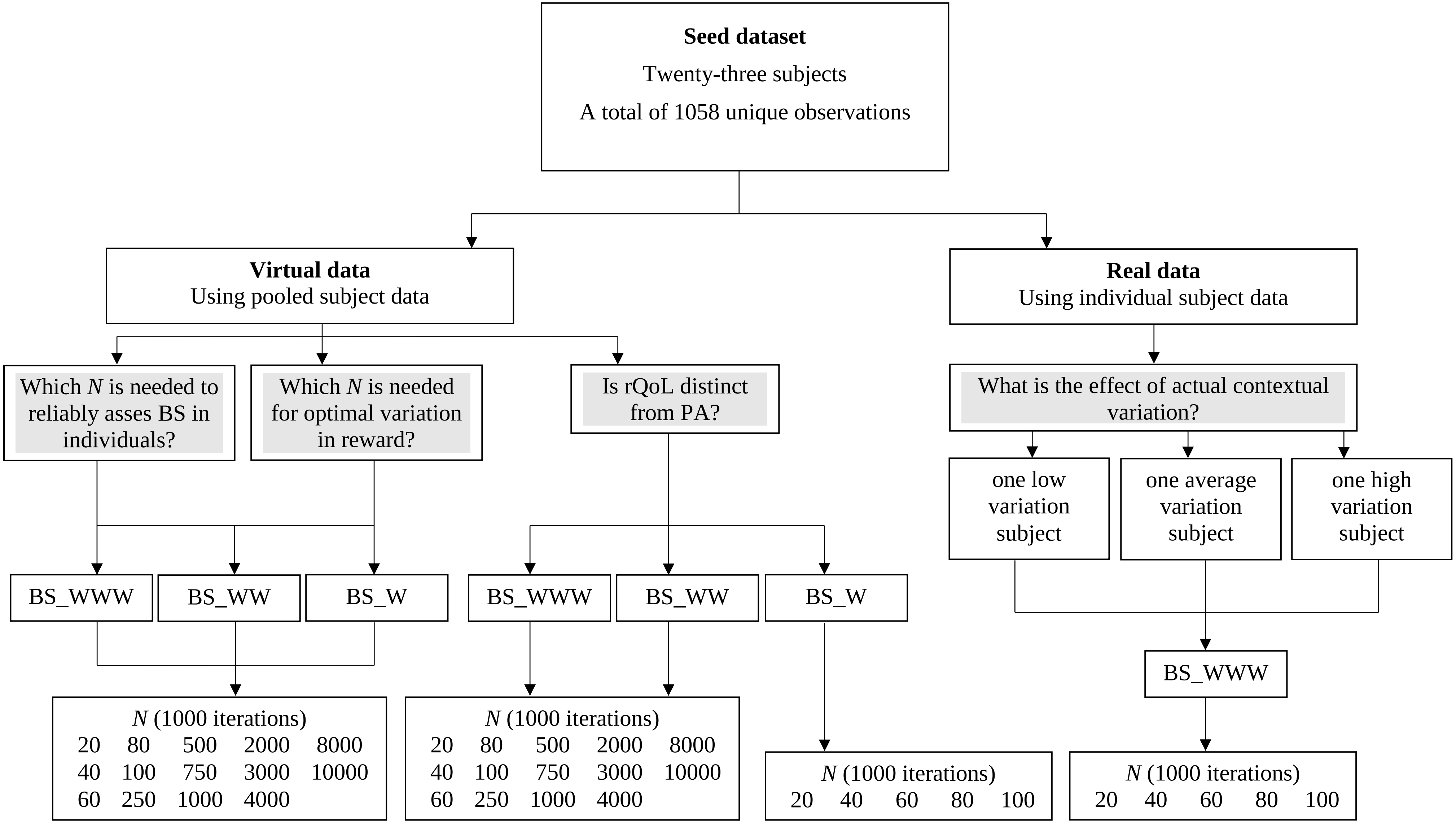

Supplement: Supplementary file 1 [file Presentation1.pdf]
